# Suppression of nbe‐miR166h‐p5 attenuates leaf yellowing symptoms of potato virus X on *Nicotiana benthamiana* and reduces virus accumulation

**DOI:** 10.1111/mpp.12717

**Published:** 2018-09-28

**Authors:** Shu Wang, Weijun Cui, Xinyang Wu, Quan Yuan, Jinping Zhao, Hongying Zheng, Yuwen Lu, Jiejun Peng, Lin Lin, Jianping Chen, Fei Yan

**Affiliations:** ^1^ College of Agriculture and Biotechnology Zhejiang University Hangzhou 310058 China; ^2^ The State Key Laboratory Breeding Base for Sustainable Control of Pest and Disease, Key Laboratory of Biotechnology in Plant Protection of MOA of China and Zhejiang Province, Institute of Virology and Biotechnology Zhejiang Academy of Agricultural Sciences Hangzhou 310021 China; ^3^ College of Plant Protection Northwest A & F University Yangling 712100 China; ^4^ Institute of Plant Virology Ningbo University Ningbo 315211 China

**Keywords:** chlorophyll, leaf yellowing, miRNA, nbe‐miR166h‐p5, *Potato virus X*

## Abstract

MicroRNAs (miRNAs) play essential roles in plant development. There is increasing evidence that changed expression of miRNAs in virus‐infected plants contributes to the development of viral symptoms. Here, we analysed the altered expression of miRNAs of *Nicotiana benthamiana* in response to *Potato virus X* (PVX) by Illumina Solexa sequencing. One of the 21 miRNAs significantly affected, nbe‐miR166h‐p5, was closely associated with viral symptoms. Using the *Tobacco rattle virus*‐based miRNA suppression (VbMS) system, we found that the suppression of nbe‐miR166h‐p5 in plants caused leaves to turn dark green with increased chlorophyll. When PVX was inoculated on nbe‐miR166h‐p5‐suppressed plants, the leaf yellowing symptom of PVX was largely attenuated with less reduction in chlorophyll content, and the accumulation of PVX was decreased. nbe‐miR166h‐p5 was also up‐regulated in plants infected by *Turnip mosaic virus* (TuMV), and its suppression attenuated the leaf yellowing symptom of TuMV and decreased viral accumulation. Three potential targets of nbe‐miR166h‐p5 were identified. The results indicate the association of nbe‐miR166h‐p5 with symptoms of PVX and also with those of TuMV, providing useful information on the relationship between miRNA and viral infection.

## Introduction

MicroRNAs (miRNAs) are small, 19–24‐nucleotide RNAs that play essential roles in eukaryotes by targeting complementary mRNAs for degradation or translational repression (Bologna and Voinnet, 2014; Cui *et al*., 2017). In plants, miRNAs regulate leaf morphogenesis, the development of roots and flowers, and other key processes (Chen, 2004; Guo *et al*., 2005; Jones‐Rhoades *et al*., 2006; Kidner and Martienssen, 2005; Palatnik *et al*., 2003; Wang *et al*., 2005). They also function in plant defence against pathogens, either by targeting the resistance gene of the plant or the pathogenesis gene of the pathogen, or by regulating the resistance pathway (Jin, 2008; Katiyar‐Agarwal and Jin, 2010; Li *et al*., 2012; Llave, 2004; Lu *et al*., 2008; Navarro *et al*., 2006; Padmanabhan *et al*., 2009; Ruiz‐Ferrer and Voinnet, 2009; Shivaprasad *et al*., 2012; Zhang *et al*., 2006).

Viruses are economically important plant pathogens that typically cause symptoms of yellowing, mosaic, twisting and even necrosis of leaves, and plant death in many cases. Studies with many different viruses have shown that virus‐infected plants generally have a changed expression profile of miRNAs (Abreu *et al*., 2014; Amin *et al*., 2011; Bazzini *et al*., 2011; Chen *et al*., 2012, 2016; Cillo *et al*., 2009; Du *et al*., 2011; Feng *et al*., 2009, 2014; Guo *et al*., 2012; Lang *et al*., 2011a, 2011b; Liu *et al*., 2015; Naqvi *et al*., 2010; Pradhan *et al*., 2015; Sun *et al*., 2015; Wang *et al*., 2015; Xu *et al*., 2014; Yang *et al*., 2016) and, in some cases, these changes are associated with viral symptoms (Gao *et al*., 2013; Lian *et al*., 2016; Romanel *et al*., 2012). Bazzini et al. (2007) reported that infection by various tobamoviruses, potyviruses and potexviruses changed the accumulation levels of most of the ten tested miRNAs in *Nicotiana tabacum*, and found that the viruses that produced the most severe symptoms on tobacco (*Tobacco mosaic virus* and *Tomato mosaic virus*) altered miRNA accumulation to a greater extent than viruses that produced mild symptoms [*Tobacco etch virus* and *Potato virus Y* (PVY)]. A correlation between symptom severity and levels of certain miRNAs also occurred in experiments using two synergistic viruses. Single infections produced mild symptoms, but double infection by *Potato virus X* (PVX) and either PVY or *Plum pox virus* (PPV) produced more severe symptoms in *Nicotiana benthamiana* and had a greater effect on the accumulation levels of miR156, miR171, miR398 and miR168 (Pacheco *et al*., 2012).

However, despite this well‐established correlation, there is little direct experimental evidence linking changed expression of particular miRNAs to viral symptoms (Gao *et al*., 2013; Lian *et al*., 2016; Romanel *et al*., 2012). Recently, we reported that a reduction in osa‐miR171b in *Rice stripe virus* (RSV)‐infected rice contributed to RSV symptoms (Tong *et al*., 2017). Here, we investigated the altered expression of miRNAs in PVX‐infected *N. benthamiana* through Illumina Solexa sequencing and identified an up‐regulated miRNA, named nbe‐miR166h‐p5, which was associated with a reduction in chlorophyll. Its suppression attenuated leaf yellowing on PVX‐infected plants.

## Results

### Leaf yellowing and decreased chlorophyll content of PVX‐infected *N. benthamiana*

**Figure 1:**
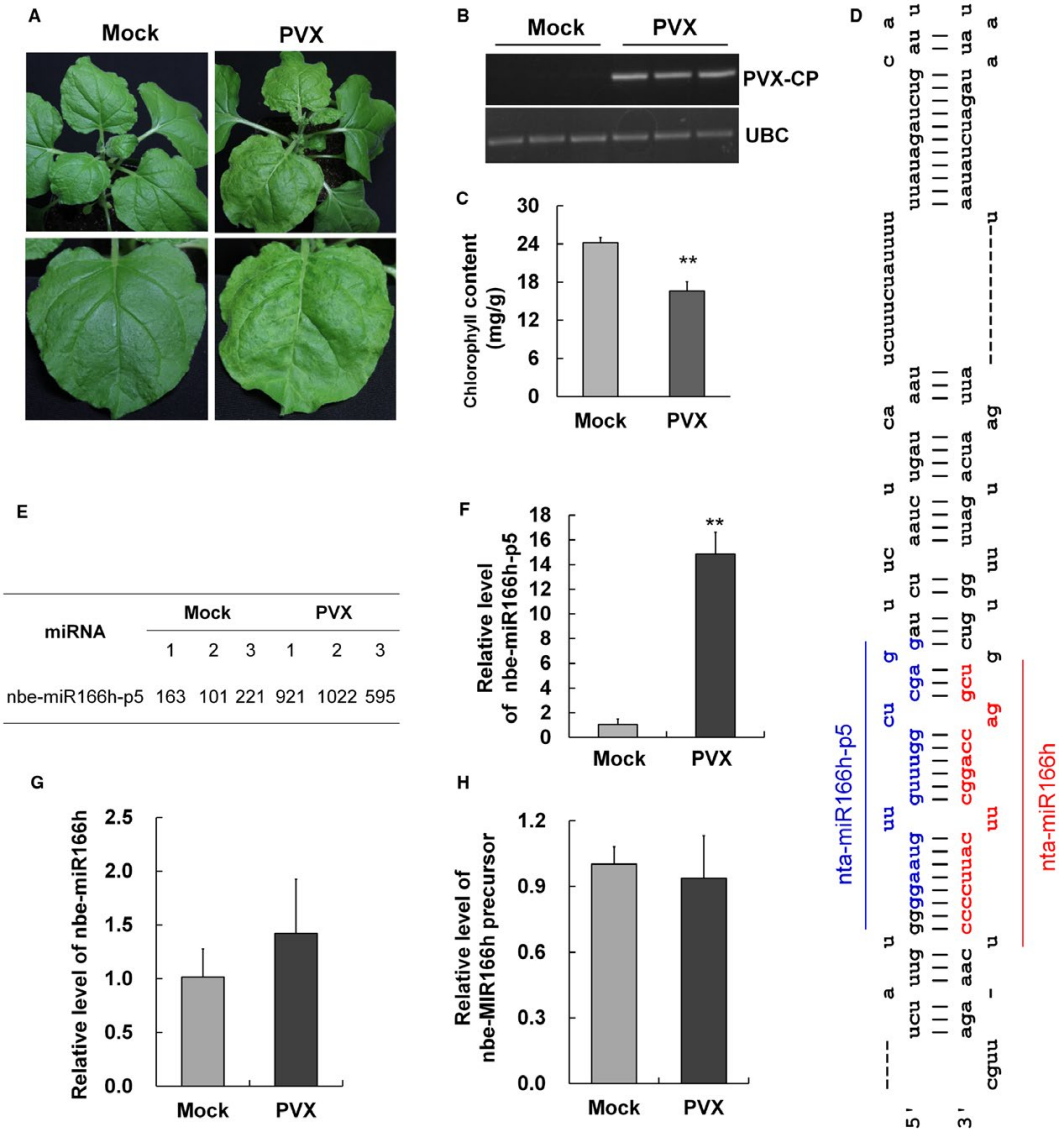
*Potato virus X* (PVX) symptoms and the altered expression of nbe‐miR166h‐p5 in PVX‐infected plants. (A) PVX symptoms were fully developed on leaves of plants at 8 days post‐inoculation (dpi) with yellowing and slight twisting. (B) Reverse transcription‐polymerase chain reaction (RT‐PCR) confirmed infection with PVX with primers designed from the PVX coat protein gene (*PVX‐CP*). The Ubiquitin C gene (*UBC*) was used as the internal reference gene to show that RNAs from mock‐inoculated plants were not degraded or absent. (C) The chlorophyll content in PVX‐infected leaves was significantly less than that in mock leaves. (D) The predicted secondary structure of the precursor of nta‐miR166h (nta‐MIR166h). The coloured sequences represent the mature nta‐miR166h and nta‐miR166h‐p5. (E) The normalized reads of nta‐miR166h‐p5 with three replicates in the Illumina Solexa sequencing data, indicating the up‐regulated expression of nta‐miR166h‐p5 in PVX‐infected plants. (F) Quantitative RT‐PCR (qRT‐PCR) demonstrating the up‐regulated expression of nta‐miR166h‐p5 in PVX‐infected plants. (G, H) qRT‐PCR showing that the expression levels of both nbe‐miR166h (G) and nbe‐MIR166h precursor (H) were not altered significantly in PVX‐infected plants. Bars represent the standard errors of the means. A two‐sample unequal variance directional *t*‐test was used to test the significance of the difference (**P < 0.01). [Colour figure can be viewed at wileyonlinelibrary.com]

At 8 days post‐inoculation (dpi) of PVX onto 3‐week‐old healthy *N. benthamiana* plants, PVX symptoms were fully developed on the upper leaves which showed yellowing and slight twisting (Fig. 1A). PVX RNAs were detectable in these symptomatic leaves, but not in those of mock‐inoculated control plants (Fig. 1B). As expected, the yellowed leaves contained significantly less chlorophyll than the controls (Fig. 1C).

### Altered expression of miRNAs in PVX‐infected *N. benthamiana* leaves

Illumina Solexa sequencing was then used to analyse the global response of miRNAs to PVX infection. Samples of the top leaves were collected from three separate plants of both inoculated and control treatments at 6 dpi, when the systemic symptoms began to appear. After removal of the junk, adapter and repeat reads, more than three million unique clean reads were obtained from each of the six libraries. An overview of the deep sequencing results is presented in Table 1.

**Table 1:** Overview of the deep sequencing data.

	PVX1	PVX2	PVX3	Mock1	Mock2	Mock3
Raw reads	11 110 037	12 235 011	10 307 223	14 270 284	14 445 774	13 100 005
Uniq raw reads	5 646 189	6 619 686	5 223 220	5 445 626	5 500 181	5 310 888
Clean reads	7 010 635	8 969 507	6 604 080	6 810 281	6 972 374	6 186 027
Uniq clean reads	4 240 384	5 203 739	3 982 951	3 829 266	3 801 670	3 793 954

A total of 2554 small RNAs was identified to have the same sequence as miRNAs from 67 different plant species, indicating the conservation of miRNAs among different plants (Table S1, see Supporting Information). From *N. tabacum*, 164 miRNAs belonging to 53 families have been deposited in the miRNA database (https://www.mirbase.org). Our *N. benthamiana* libraries contained homologues of 86 of these *N. tabacum* miRNAs without mismatch in sequence (Table S2, see Supporting Information). Of these 86 conserved miRNAs, 21 showed significantly altered expression (*P *< 0.05) after PVX infection, 17 being up‐regulated and four down‐regulated (Table 2).

**Table 2:** Twenty‐one microRNAs (miRNAs) that were conserved in *Nicotiana tabacum* and *Nicotiana benthamiana* and that showed significantly altered expression (*P *< 0.05) after *Potato virus X* (PVX) infection. Values are normalized reads.

miRNA name	miRNA sequence	PVX1	PVX2	PVX3	Mock1	Mock2	Mock3
nta‐MIR396a‐p3	GCTGTGGGAAGATACAGATAG	16	16	14	0	0	2
nta‐MIR6019a‐p3	ACATTTACACGTACCTTGTATG	717	833	747	165	101	127
nta‐MIR396c‐p3	GTTCAAGAAAGCTGTGGGAAA	1041	1196	1031	318	370	475
nta‐MIR6147‐p3	GTGGGTATTGAAGATGTTATG	445	596	557	103	137	158
nta‐MIR159‐p5	GAGCTCCTTGAAGTCCAACAG	4039	4184	3239	970	763	946
nta‐MIR6161a‐p3	TATTATGCTGGACCGGTATACT	652	708	602	489	454	494
nta‐MIR160c‐p3	GCGTGCGAGGAGCCAAGCATA	148	217	162	25	20	23
nta‐miR6155	TAAGGTTGCCTTGCTCTTGCA	1878	2028	2154	2395	2617	2799
nta‐MIR167d‐p3	GATCATGTGGTAGCTTCACC	2943	2101	2734	1231	706	1684
nta‐miR169a	CAGCCAAGGATGACTTGCCGA	193	240	195	116	122	111
nta‐MIR166d‐p5	GGAATGTTGTCTGGCTCGAGG	4924	5371	3169	460	383	915
nta‐miR6151f	TGAGTGTGAGGCATTGGATTGA	16	16	26	40	37	29
nta‐MIR166b‐p5	TGGCTCGAAGCCATTATCTCC	1	1	1	0	0	0
nta‐MIR166h‐p5	GGAATGTTGTTTGGCTCGAGG	921	1022	595	163	101	221
nta‐MIR172d‐p5	GTAGCATCATCAAGATTCACA	1727	2359	1782	940	1328	1036
nta‐miR168d	TCGCTTGGTGCAGGTCGGGAA	1304	1769	1294	821	662	992
nta‐miR6153	TAGGACCATATTCACTATTTG	681	807	938	1008	1280	1126
nta‐MIR166g‐p5	AGAATGTTGTCTGGTTCGTGA	15	20	11	4	3	5
nta‐miR6151a	TGAATGTGAGGCATTGGATTGA	0	1	1	4	2	5
nta‐MIR171c‐p5	TATTGGCCTGGTTCACTCAGA	1258	1789	1209	729	556	763
nta‐MIR160a‐p3	GCGTATGAGGAGCCAAGCATA	1143	1899	1012	110	114	143

### Suppression of nbe‐miR166h‐p5 attenuates the leaf yellowing symptoms of PVX

One of the miRNAs that was up‐regulated in PVX‐infected *N. benthamiana* plants was nbe‐miR166h‐p5, a homologue of *N. tabacum* miR166h‐p5 (nta‐miR166h‐p5) with no mismatch in sequence. It is known that nta‐miR166h‐p5 is produced from the 5′ end of the precursor nta‐MIR166h and is the miRNA* of nta‐miR166h (Fig. 1D). The predicted precursor of nbe‐miR166h was exactly the same as that of nta‐MIR166h, indicating the conservation of this miRNA (Fig. S1, see Supporting Information). The changed expression level of nbe‐miR166h‐p5 in PVX‐infected plants was confirmed by quantitative reverse transcription‐polymerase chain reaction (qRT‐PCR), which indicated that it was up‐regulated about 14‐fold (Fig. 1F). In further experiments, it was found that the expression levels of the complementary nbe‐miR166h and the precursor nbe‐MIR166h were not altered significantly in PVX‐infected plants (Fig. 1G,H). This possibly indicates that additional nbe‐miR166h‐p5 was specifically produced from its precursor in PVX‐infected plants, or that the steady‐state level of nbe‐miR166h‐p5 was enhanced in PVX‐infected plants.

**Figure 2:**
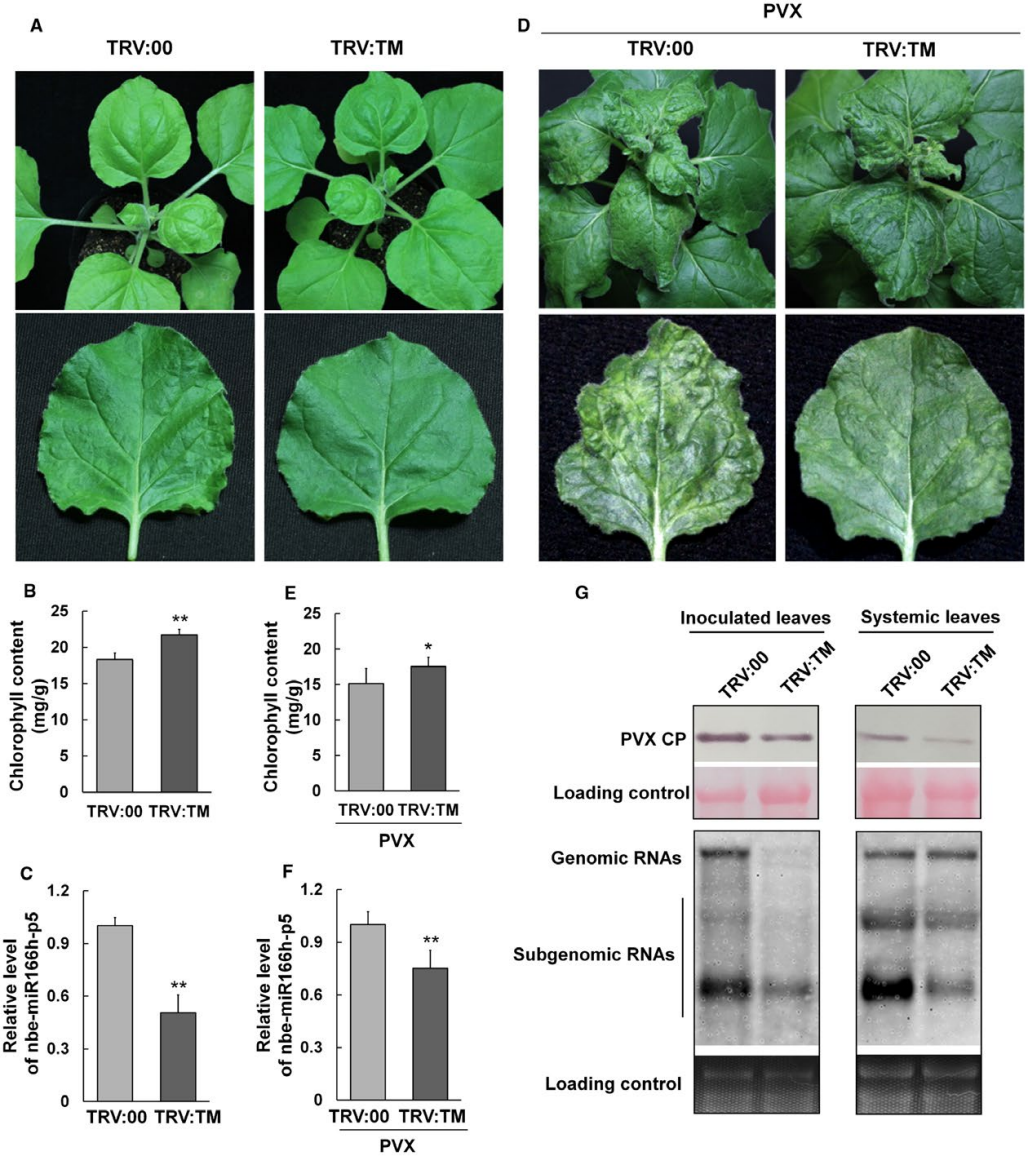
Suppression of nbe‐miR166h‐p5 attenuates the leaf yellowing symptoms of *Potato virus X* (PVX) and reduces viral accumulation. (A) A *Tobacco rattle virus* (TRV)‐based microRNA (miRNA) suppression (VbMS) system was used to suppress the expression of nbe‐miR166h‐p5 (TRV:TM). At 14 days post‐inoculation (dpi), the phenotype of the TRV:TM‐treated plant was similar to that of the control (TRV:00), but the leaves were dark green. (B) The content of chlorophyll in TRV:TM‐treated leaves was significantly higher than that in control leaves. (C) Quantitative reverse transcription‐polymerase chain reaction (qRT‐PCR) showed that the expression level of nbe‐miR166h‐p5 in TRV:TM‐treated plants was reduced to approximately 50% of that in control plants. (D) PVX was inoculated onto TRV:TM‐treated and control plants 14 days after VbMS treatment. At 8 dpi of PVX, only a small area of yellowing appeared on nbe‐miR166h‐p5‐suppressed leaves (TRV:TM), whereas, on leaves of the control plants, there were typical PVX symptoms with much more yellowing (TRV:00). (E) The chlorophyll content in nbe‐miR166h‐p5‐suppressed leaves was significantly higher than that in control leaves. (F) qRT‐PCR showed that the level of nbe‐miR166h‐p5 in PVX‐infected TRV:TM‐treated plants was approximately 70% of that in PVX‐infected TRV:00‐treated plants. (G) Western blotting and northern blotting showed that PVX accumulated at a lower level in both inoculated leaves and systemic leaves of nbe‐miR166h‐p5‐suppressed plants. CP, coat protein. Bars represent the standard errors of the means. A two‐sample unequal variance directional *t*‐test was used to test the significance of the difference (*P < 0.05; **P < 0.01). [Colour figure can be viewed at wileyonlinelibrary.com]

To investigate the association of nbe‐miR166h‐p5 with PVX infection, the expression of this miRNA was suppressed using the *Tobacco rattle virus* (TRV)‐based miRNA suppression (VbMS) system (Yan *et al*., 2014), and the progress of PVX infection was monitored. For VbMS, a target mimic (TM) sequence that would sequester nbe‐miR166h‐p5 was designed according to the methods described previously and introduced into the TRV vector, producing TRV:TM (Yan *et al*., 2014). TRV:TM or TRV:00 (empty vector control) was inoculated onto plants by agroinfiltration. Fourteen days later, the only obvious difference in phenotype between TRV:TM and control plants was that leaves of TRV:TM‐treated plants were darker green (Fig. 2A). The chlorophyll content of TRV:TM‐treated leaves was significantly higher than in control leaves (Fig. 2B). Using qRT‐PCR, it was confirmed that the expression of nbe‐miR166h‐p5 was suppressed in VbMS‐treated plants, to approximately 50% of its level in control plants (Fig. 2C). Further experiments showed that there was no statistically significant change in expression of nbe‐miR166h in VbMS‐treated plants (Fig. S2, see Supporting Information).

Next, PVX was inoculated onto TRV:TM‐treated and control plants after 14 days of VbMS treatment. Eight days later, control plants showed typical yellowing symptoms of PVX, but there was only a small area of yellowing on the leaves of nbe‐miR166h‐p5‐suppressed plants (Fig. 2D). Correspondingly, the chlorophyll content of nbe‐miR166h‐p5‐suppressed leaves was significantly higher than that in control leaves (Fig. 2E). At this time point, the expression level of nbe‐miR166h‐p5 in PVX‐infected TRV:TM‐treated plants was approximately 70% of that in PVX‐infected TRV:00‐treated plants, indicating that nbe‐miR166h‐p5 was still suppressed under PVX infection (Fig. 2F). Taken together, these results indicate that the suppression of nbe‐miR166h‐p5 attenuates the leaf yellowing symptoms of PVX in *N. benthamiana*.

### Suppression of nbe‐miR166h‐p5 reduces the accumulation of PVX in *N. benthamiana* plants

We also detected the accumulation levels of PVX in nbe‐miR166h‐p5‐suppressed plants. At 8 dpi, both inoculated leaves and upper systemically infected leaves were sampled and used in western blotting to detect the PVX coat protein (CP) and in northern blotting to detect the viral genomic and subgenomic RNAs. PVX CP accumulated at lower levels in both inoculated and systemic leaves of suppressed plants (Fig. 2G, top part). In inoculated leaves, both PVX genomic and subgenomic RNAs were reduced, whereas, in systemic leaves, PVX genomic RNAs were not significantly reduced, but subgenomic RNAs accumulated at a much lower level compared with those in inoculated leaves (Fig. 2G, bottom part). These results suggest that the suppression of nbe‐miR166h‐p5 reduces the accumulation of PVX in *N. benthamiana* plants and, particularly, the accumulation of subgenomic RNAs in systemic leaves.

**Figure 3:**
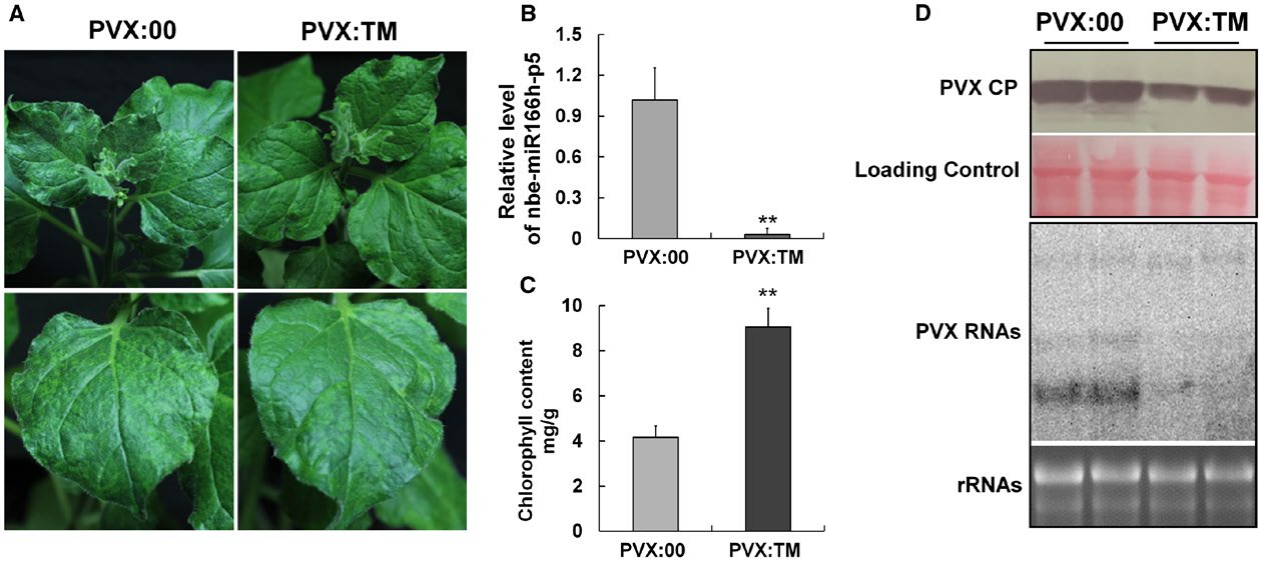
The leaf yellowing symptom was also attenuated when nbe‐miR166h‐p5 was suppressed by *Potato virus X* (PVX)‐mediated virus‐based microRNA (miRNA) suppression (VbMS). (A) PVX itself was used as a tool to suppress nbe‐miR166h‐p5 through a VbMS system. At 8 days post‐inoculation (dpi), nbe‐miR166h‐p5‐suppressed plants (PVX:TM) showed attenuated symptoms compared with the control (PVX:00). (B) Quantitative reverse transcription‐polymerase chain reaction (qRT‐PCR) showed that suppressed plants (PVX:TM) had 5% of the level of nbe‐miR166h‐p5 present in control plants (PVX:00). (C) The chlorophyll content in nbe‐miR166h‐p5‐suppressed leaves (PVX:TM) was significantly higher than that in control leaves (PVX:00). (D) Western and northern blotting showed that PVX accumulated at a lower level in both inoculated and systemic leaves in nbe‐miR166h‐p5‐suppressed plants. CP, coat protein. Bars represent the standard errors of the means. A two‐sample unequal variance directional *t*‐test was used to test the significance of the difference (**P < 0.01). [Colour figure can be viewed at wileyonlinelibrary.com]

PVX itself has also been developed as a tool for VbMS and has a much stronger effect than TRV in suppressing miRNAs (Zhao *et al*., 2016). Hence, we also used the PVX‐mediated VbMS system to analyse the effect of suppression of nbe‐miR166h‐p5 on PVX accumulation. At 8 dpi, plants treated with PVX:TM (the target mimic) or PVX:00 (empty vector control) both showed viral symptoms, but the symptoms on PVX:TM plants were much milder than those on plants treated with PVX:00 (Fig. 3A). The expression of nbe‐miR166h‐p5 in VbMS‐treated plants was suppressed to 5% of the level in control plants (Fig. 3B), and the chlorophyll content of nbe‐miR166h‐p5‐suppressed leaves was significantly higher than that of the controls (Fig. 3C). Both the PVX CP and viral RNAs in PVX:TM‐treated plants were much lower than those in PVX:00‐treated plants (Fig. 3D). These results are consistent with those from TRV‐mediated VbMS, further indicating the association of nbe‐miR166h‐p5 with PVX symptoms.

### Suppression of nbe‐miR166h‐p5 also attenuates symptoms and reduces viral accumulation of *Turnip mosaic virus* (TuMV) on *N. benthamiana*

**Figure 4:**
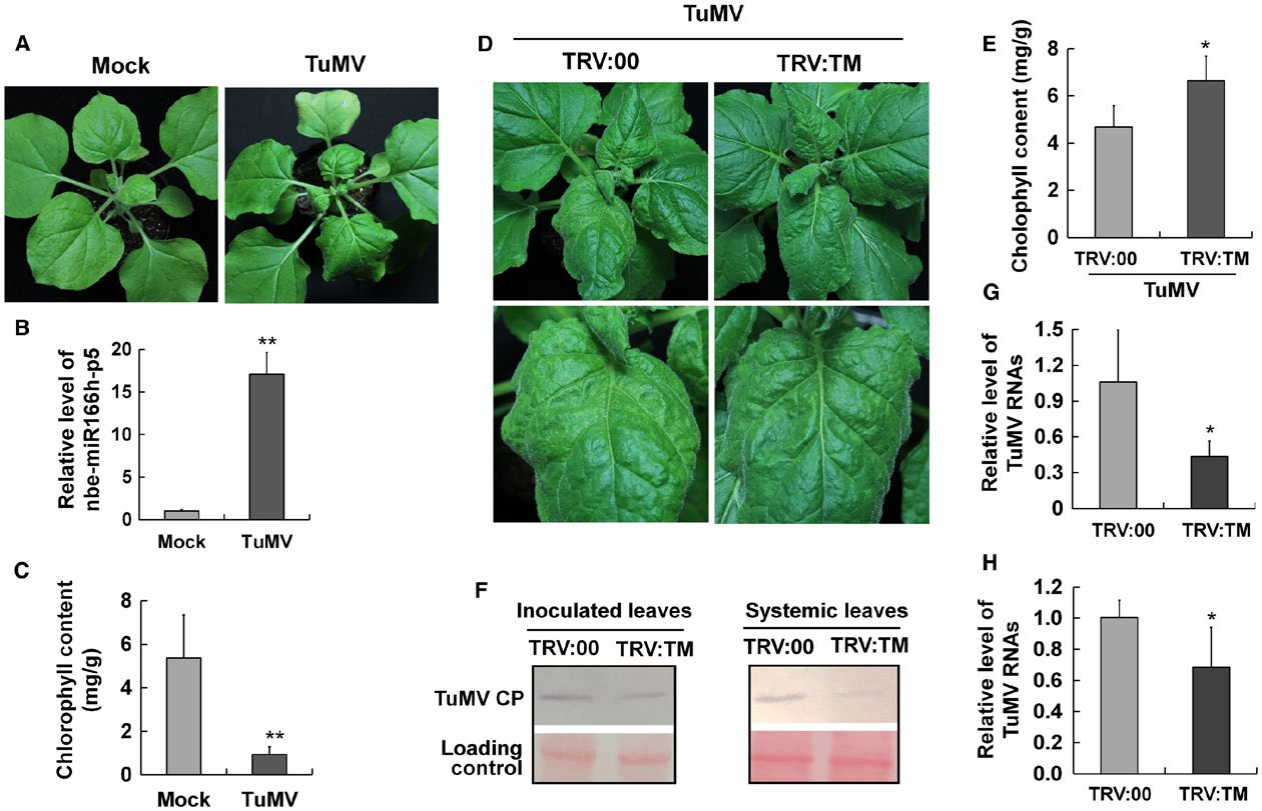
Suppression of nbe‐miR166h‐p5 attenuated the symptoms of *Turnip mosaic virus* (TuMV) on *Nicotiana* *benthamiana* and reduced viral accumulation. (A) TuMV symptoms at 8 days post‐inoculation (dpi). (B) Quantitative reverse transcription‐polymerase chain reaction (qRT‐PCR) showed the up‐regulated expression of nbe‐miR166h‐p5 in TuMV‐infected plants. (C) The chlorophyll content in TuMV‐infected leaves was significantly less than that in mock leaves. (D) TuMV was inoculated onto TRV:TM‐treated and control plants at 14 days after virus‐based microRNA (miRNA) suppression (VbMS) treatment. At 8 dpi of TuMV, the leaf yellowing symptom was mild on leaves of nbe‐miR166h‐p5‐suppressed plants compared with that on control plants. (E) The chlorophyll content in nbe‐miR166h‐p5‐suppressed leaves was significantly higher than that in control leaves. (F) Western blotting showed that TuMV accumulated at a lower level in both inoculated and systemic leaves of nbe‐miR166h‐p5‐suppressed plants. CP, coat protein. (G, H) qRT‐PCR showed that TuMV RNAs accumulated at a lower level in both inoculated leaves (G) and systemic leaves (H) of nbe‐miR166h‐p5‐suppressed plants. Bars represent the standard errors of the means. A two‐sample unequal variance directional *t*‐test was used to test the significance of the difference (*P < 0.05; **P < 0.01). [Colour figure can be viewed at wileyonlinelibrary.com]

To investigate whether nbe‐miR166h‐p5 was associated in a similar way with the symptoms of a different virus, we conducted similar experiments with the potyvirus TuMV. At 8 dpi, when TuMV symptoms of leaf yellowing and distortion were fully developed, the expression of nbe‐miR166h‐p5 was up‐regulated by approximately 16‐fold compared with mock‐inoculated plants (Fig. 4A, B). The chlorophyll content in TuMV‐infected leaves was also decreased significantly (Fig. 4C). When TuMV was inoculated onto TRV:TM‐treated plants with nbe‐miR166h‐p5 suppression, the leaf yellowing symptoms were milder at 8 dpi than on control plants (Fig. 4D). Correspondingly, the chlorophyll content in nbe‐miR166h‐p5‐suppressed leaves was significantly higher than that in control leaves (Fig. 4E). TuMV CP and viral RNA levels were also much lower in TRV:TM‐treated plants than in the controls (Fig. 4F–H). These results indicate that the suppression of nbe‐miR166h‐p5 also attenuates the symptoms and reduces the viral accumulation of TuMV on *N. benthamiana*.

### Targets of nbe‐miR166h‐p5 in *N. benthamiana* plants

**Figure 5:**
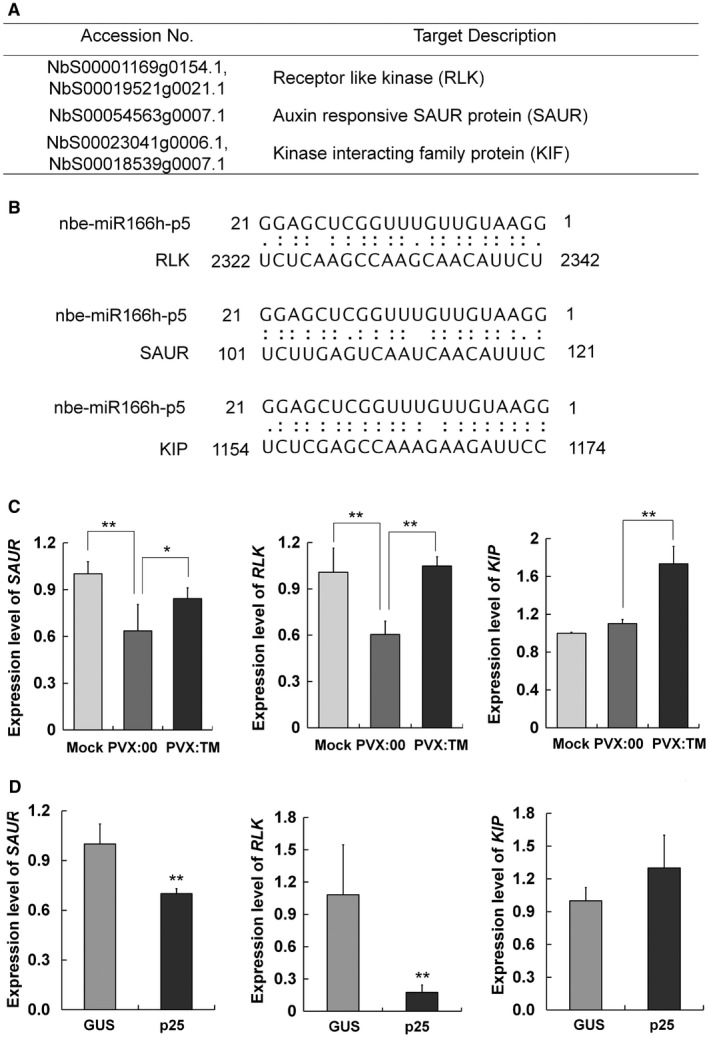
Targets of nbe‐miR166h‐p5 in *Nicotiana benthamiana* plants. (A) Three targets were predicted through psRNATarget (https://plantgrn.noble.org/psRNATarget/?function=1). (B) The binding site of nbe‐miR166h‐p5 on the mRNAs of each potential target was localized at the encoding sequence. (C) Quantitative reverse transcription‐polymerase chain reaction (qRT‐PCR) showed that the mRNA levels of *RLK* and *SAUR* were down‐regulated in *Potato virus X* (PVX)‐infected plants (PVX). Meanwhile, the mRNA levels of the three targets were up‐regulated in nbe‐miR166h‐p5‐suppressed plants (PVX:TM) relative to PVX‐infected plants. (D) qRT‐PCR showed that the mRNA levels of *RLK* and *SAUR* were down‐regulated in p25‐expressed leaves, whereas the mRNA levels of *KIP* were not altered significantly. In the experiment, p25 and the unrelated β‐glucuronidase (GUS) protein (as control) were transiently expressed in leaves through agroinfiltration. The infiltrated leaves were collected for analysis at 3 days post‐inoculation (dpi). Bars represent the standard errors of the means. A two‐sample unequal variance directional *t*‐test was used to test the significance of the difference (**P < 0.01).

The function of miRNAs depends on the targeting of their complementary mRNAs for degradation or translational repression. The potential targets of nbe‐miR166h‐p5 have not been reported previously. Using psRNATarget (https://plantgrn.noble.org/psRNATarget/?function=1), three potential targets of nbe‐miR166h‐p5 were predicted with expectation <2.5: encoding receptor‐like kinase (RLK), auxin‐responsive SAUR protein and kinase interacting family protein (KIP) (Fig. 5A). The binding site of nbe‐miR166h‐p5 on the mRNAs of each potential target was localized at the encoding sequence (Fig. 5B). The mRNA levels of these three predicted targets were all up‐regulated in nbe‐miR166h‐p5‐suppressed plants (Fig. 5C). Consistent with these findings, the mRNA levels of RLK and SAUR were down‐regulated in PVX‐infected plants, which was expected because of the higher expression of nbe‐miR166h‐p5 (Fig. 5C). Meanwhile, the mRNA level of KIP in PVX‐infected plants was not altered significantly (Fig. 5C). We suppose that there may be other factors balancing the expression of this target. The expression levels of the three targets in leaves expressing p25, the PVX suppressor of RNA silencing, were also determined and similar results were obtained, indicating that p25 may play a role in the regulation of nbe‐miR166h and the expression of its targets (Fig. 5D).

**Figure 6:**
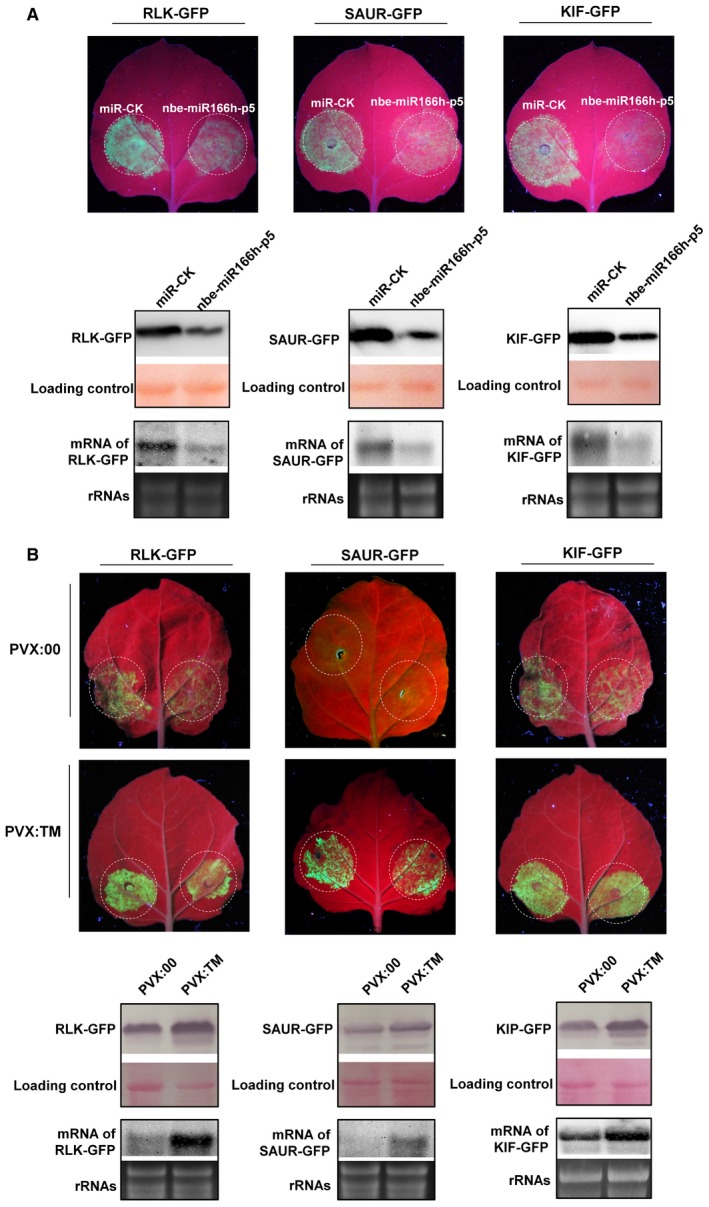
Interaction of nbe‐miR166h‐p5 with the three predicted target mRNAs. (A) Green fluorescent protein (GFP)‐fused RLK, SAUR and KIF were expressed at a low level when co‐expressed with nbe‐miR166h‐p5, but at a high level when co‐expressed with a control microRNA (miRNA) (miR‐CK). (B) Differences in transient expression of GFP‐fused RLK, SAUR and KIP between PVX:TM‐treated and PVX:00‐treated plants. GFP fluorescence was much more intense on PVX:TM‐treated than on PVX:00‐treated plants. The green fluorescence, protein and mRNA level of GFP‐fused targets are shown. [Colour figure can be viewed at wileyonlinelibrary.com]

To examine the interaction of nbe‐miR166h‐p5 with these three predicted target mRNAs, the three genes were cloned and fused with green fluorescent protein (GFP); they were then expressed by agroinfiltration, together with nbe‐miR166h‐p5 expressed by artificial miRNA, as described previously (Shi *et al*., 2016). At 3 dpi, GFP‐fused RLK, SAUR and KIF were expressed at a low level when co‐expressed with nbe‐miR166h‐p5, but at a high level when co‐expressed with a control miRNA (miR‐CK) (Fig. 6A). The mRNAs of GFP‐fused RLK, SAUR and KIP were also significantly decreased in cells co‐expressing nbe‐miR166h‐p5 (Fig. 6A). We also investigated the differences in transient expression of GFP‐fused RLK, SAUR and KIP between PVX:TM‐treated and PVX:00‐treated plants. GFP fluorescence on PVX:TM‐treated plants was much more intense than that on PVX:00‐treated plants (Fig. 6B), and the mRNAs and proteins of GFP‐fused RLK, SAUR and KIP accumulated to a more significant extent in PVX:TM‐treated plants (Fig. 6B). Taken together, these results confirm that RLK, SAUR and KIP could be targeted by nbe‐miR166h‐p5 for regulation.

As the suppression of nbe‐miR166h‐p5 increased the content of chlorophyll in plants, it seems reasonable to suppose that silencing of the targets might have the opposite effect. We therefore silenced RLK, SAUR and KIP individually using the TRV‐induced gene silencing system, and determined the chlorophyll content in silenced plants. No obvious phenotype was visible on silenced plants and there was no significant difference in chlorophyll content (Fig. S3, see Supporting Information).

## Discussion

miRNAs play key roles in plant development and defence against pathogens. Infection by many different viruses changes the level of certain miRNAs in plants, and it is supposed that the altered miRNA profile results in viral symptoms. For PVX, it has been reported that miR160, miR164, miR166, miR168, miR169 and miR171 are up‐regulated in PVX‐infected *N. tabacum* (Bazzini *et al*., 2007; Lang *et al*., 2011a) and, of the ten tested miRNAs in PVX‐infected *N. benthamiana*, miR156, miR171, miR398 and miR168 showed altered expression levels (Pacheco *et al*., 2012). These results predate the availability of data based on high‐throughput small RNA sequencing which we have used in this study to provide a more comprehensive survey of the miRNAs that respond to PVX in *N. benthamiana*. Twenty‐one miRNAs were identified to show significant changes in expression in PVX‐infected *N. benthamiana* at 6 dpi. Of these 21 miRNAs, miR168, miR169 and miR171 have been reported previously to be regulated in PVX‐infected *N. tabacum* or *N. benthamiana.* The other miRNAs identified in previous studies (miR156, miR160, miR164, miR166 and miR398) did not show obviously or significantly changed expression levels in our experiments. This difference may be because our samples were collected at 6 dpi when symptoms began to appear on the top leaves of plants, which was earlier than in the previously reported studies. More detailed data are needed to understand how individual miRNA expression levels change during the time course of PVX infection.

miR166h‐p5 is produced from the 5′ arm of the MIR166h precursor and is significantly up‐regulated in PVX‐infected *N. benthamiana* plants. This miRNA has not been reported previously to respond to viral infection, although miR166h, produced from the 3′ arm of the MIR166 precursor, shows changed expression in *Potato spindle tuber viroid* (PSTVd)‐infected *Phelipanche ramose* and *Mungbean yellow mosaic India virus* (MYMIV)‐infected *Vigna mungo* (Ivanova *et al*., 2014; Kundu *et al*., 2017). In a previous study, it has been reported that RSV infection induces the expression of novel miRNAs in a phased pattern from several conserved miRNA precursors, and enhances the accumulation of some rice miRNA*s, but not their corresponding miRNAs (Du *et al*., 2011). In this study, more nbe‐miR166h‐p5 was specifically produced from its precursor in PVX‐infected plants, whereas the expression of the precursor and nbe‐miR166h was not affected. Notably, the mRNA levels of the predicted nbe‐miR166h targets were down‐regulated in leaves expressing p25, the PVX suppressor of RNA silencing, which probably indicates that p25 might enhance the level of nbe‐miR166h‐p5 to regulate its targets. This is worthy of further study. We also investigated whether other small RNAs were produced from the nbe‐MIR166h precursor, but did not find any (data not shown), indicating that the phased miRNA was not produced from the nbe‐MIR166h precursor.

Suppression of nbe‐miR166h‐p5 attenuated the symptoms of PVX and enhanced the chlorophyll content (Fig. 3). Notably, the content of chlorophyll in leaves was slightly decreased when nbe‐miR166h‐p5 was transiently expressed (Fig. S4, see Supporting Information). These results suggest that nbe‐miR166h‐p5 might be involved in the regulation of chlorophyll content, and that the up‐regulated expression of nbe‐miR166h‐p5 could perhaps contribute to the leaf yellowing symptoms of PVX in *N. benthamiana* by reducing the content of chlorophyll. The results also showed that PVX RNAs accumulated at a lower level when nbe‐miR166h‐p5 was suppressed, suggesting a possible association of miRNA with resistance against PVX infection. Although we have no firm evidence as yet on how such resistance might be enhanced, we suppose that the chloroplast may possibly play a positive role. Indeed, it has been reported previously that the down‐regulation of the *psaK* gene, encoding a chloroplastic protein, leads to higher PPV accumulation, indicating the positive role of the chloroplast against viral infection (Jimenez *et al*., 2006). Further studies are needed to demonstrate whether the increased accumulation of chlorophyll in nbe‐miR166h‐p5‐suppressed plants might strengthen the defence function of chloroplasts, hence increasing their resistance to PVX.

We identified three targets of nbe‐miR166h‐p5 in *N. benthamiana*: RLK, auxin‐responsive SAUR protein and KIP. RLKs are transmembrane proteins with putative amino‐terminal extracellular domains and carboxyl‐terminal intracellular kinase domains in plants. They control a wide range of processes, including development, disease resistance, hormone perception and self‐incompatibility (Plant receptor‐like kinase gene family: diversity, function and signalling). Auxin‐responsive SAUR protein is encoded by a group of small, auxin‐induced RNAs. The function of SAUR proteins is still unknown; however, they may play some role in an auxin signal transduction pathway that involves calcium and calmodulin (Auxin‐responsive gene expression: genes, promoters and regulatory factors). KIP functions for kinase through interaction. Members of the RLK, SAUR and KIP families are abundant in plants and it is possible that they may complement the functions of other family members when one is silenced, which might help to explain why the silencing of the three identified targets did not affect the chlorophyll content. Although no direct effect of silencing of these targets on chlorophyll content was detected here, we could not exclude an indirect role of these targets on the regulation of chlorophyll, which is worthy of future study. In particular, the overexpression of these targets in plants could provide more useful information to understand the relationships between nbe‐miR166h‐p5, its targets and chlorophyll content.

Abnormal chloroplast and chloroplast‐related components are associated with viral symptoms. It has been well reported that the expression of genes implicated in photosynthesis and the components participating in photosynthesis is reduced in different virus‐infected plants (Baumgartnerova *et al*., 1998; Funayama *et al*., 1997; Lehto *et al*., 2003; Liu *et al*., 2014; Lu *et al*., 2012; Rodriguez *et al*., 2012). In a previous report, we have provided evidence that the down‐regulation of chloroplast‐related genes (ChRGs) contributes to RSV‐induced chlorosis or yellowing (Shi *et al*., 2016). It is known that small RNAs can regulate ChRGs directly. A small interfering RNA (siRNA) derived from *Cucumber mosaic virus* (CMV) satellite RNA determines the viral yellowing symptoms by targeting and silencing the host *magnesium protoporphyrin chelatase subunit* I gene (*Chl*I, the key gene involved in chlorophyll synthesis) (Shimura *et al*., 2011; Smith *et al*., 2011). Here, we demonstrated that nbe‐miR166h‐p5 might function by regulating the content of chlorophyll, thus participating in PVX symptom development. However, such regulation would have to be indirect, as no ChRGs were identified as potential targets of nbe‐miR166h‐p5, and silencing of its targets was not sufficient to affect chlorophyll content. Further studies are needed to understand how nbe‐miR166h‐p5 reduces the content of chlorophyll.

In conclusion, our results provide an insight into the response of miRNAs to PVX in *N. benthamiana*, demonstrating that suppression of nbe‐miR166h‐p5 attenuates leaf yellowing symptoms and reduces virus accumulation.

## Experimental procedures

### Plants and virus inoculation

*Nicotiana benthamiana* plants were grown in a glasshouse at 23–25 °C, with a 12‐h day/night light cycle under well‐watered conditions. Three‐week‐old plants were used for mechanical inoculation with viruses. After symptoms appeared on the upper leaves of plants, leaves were collected from three independent infected plants as three replicates for analysis.

### VbMS vector construction

VbMS vectors were constructed by *in vitro* RNA transcription using KOD polymerase (Toyobo, Osaka, Japan), and the synthesized DNA templates were prepared by PCR with a pair of primers (STTM166h‐p5‐FP and STTM166h‐p5‐RP) and the DNA template 48‐nucleotide spacer (Table S3, see Supporting Information). The amplified fragment was ligated into TRV‐RNA2 or the PVX vector through the Lic sequence according to the method described previously (Zhao *et al*., 2016). TRV‐RNA2 and PVX were digested with *Pst*I and *Sma*I, respectively. The digested vector and PCR product were treated with T4 DNA polymerase in the presence of dTTP and dATP. Equal volumes of vector and PCR product were mixed and incubated at 37 °C for 30 min, and then transformed into *Escherichia coli* strain DH5α. The constructs were verified by sequencing and transformed to *Agrobacterium tumefaciens* strain GV3101.

### Small RNA sequencing and analysis

Approximately 5 µg of total RNAs were isolated for the preparation of a small RNA library according to the protocol of the TruSeq Small RNA Sample Prep Kit (Illumina, California, USA). Sequencing was performed on an Illumina Hiseq2500 at LC‐BIO (Hangzhou, China) following the protocol of the manufacturer. After the removal of small RNA sequences from the 3′ adapter sequence, low quality and junk sequences, the remaining small RNA reads were collapsed to uniread sets and reads of >30 or <18 nucleotides were discarded. Clean small RNA reads were used for further bioinformatics analysis. The read numbers of small RNAs were normalized to ‘reads per million’ (RPM) based on the total small RNA read numbers. The mapping of virus‐derived siRNAs to the combined virus genome sequence was carried out according to the method described previously (Li *et al*., 2016).

### Target prediction of miRNA

Targets of nbe‐miR166h‐p5 were predicted by psRNATarget (https://plantgrn.noble.org/psRNATarget/) (Dai and Zhao, 2011). The parameter of maximum expectation was set at 2.5. The length for complementarity scoring was set at 20 bp. Target accessibility (range 0–100, less is better) was set at 25.0. The flanking length around the target site for target accessibility analysis was set at 17 bp upstream and 13 bp downstream.

### RT‐PCR and qRT‐PCR

Total RNA was isolated from plants with Trizol (Invitrogen, California, USA) according to the manufacturer’s instructions. To confirm the systemic infection of PVX, viral RNAs in a newly emerged leaf were amplified by RT‐PCR with primers designed from the PVX CP sequence, according to the method described previously (Jiang *et al*., 2014) (Table S3).

qRT‐PCR of target genes or viral RNA was performed according to a previous report (He *et al*., 2012). The *N. benthamiana* Ubiquitin C gene (*UBC*) gene (Accession Number: AB026056.1) was used as the internal reference gene for analysis. A Roche LightCycler^®^480 Real‐Time PCR System (Basel, Switzerland) was used for the reaction, and the results were analysed by the ΔΔC_T_ method. The primers used are listed in Table S3.

The qRT‐PCR for nbe‐miR166h‐p5 was performed according to a previous report (Guo *et al*., 2012). Briefly, the specific stem‐loop RT primer was used for reverse transcription from purified total RNA. The RT product was used as template for SYBR green‐based real‐time PCR analysis with the specific forward primer and the universal reverse primer. The reactions were incubated in a 384‐well plate at 95 °C for 10 min, followed by 40 cycles of 95 °C for 15 s and 60 °C for 1 min. U6 was used as the internal control. All reactions were run in triplicate, and the results were analysed by the ΔΔC_T_ method. The primers used are listed in Table S3.

### Western blotting

Total protein from plant samples was extracted and separated by 12% sodium dodecylsulphate‐polyacrylamide gel electrophoresis (SDS‐PAGE), as reported previously. After transfer onto nitrocellulose (Amersham, Uppsala, Sweden) by wet electroblotting, the proteins were detected with primary antibody to GFP or PVX and secondary anti‐mouse antibody (Sigma‐Aldrich, St Louis, USA). The antigen–antibody complexes were visualized using nitrotetrazolium blue chloride/ 5‐bromo‐4‐chloro‐3‐indolyl phosphate (NBT/BCIP) buffer (Sigma) under standard conditions.

### Measurements of chlorophyll content

Four pieces of leaf disc were collected from the tested plants. Total chlorophylls were extracted with 100% methanol, and their concentrations were determined (Lichtenthaler and Wellburn, 1983).

## Conflicts of interest

The authors declare that the research was conducted in the absence of any commercial or financial relationships that could be construed as a potential conflict of interest.

## Supporting information


**Fig. S1** Sequence alignment between the precursors of ntamiR166h and nbe‐miR166h.

**Fig. S2** The expression of nbe‐miR166h was not affected in VbMS‐treated plants.

**Fig. S3** Plants with silenced targets showed no obvious phenotype and had no significant differences in chlorophyll content.

**Fig. S4** Chlorophyll content was decreased in leaves with transient expression of nbe‐miR166h‐p5.

**Table S1** Conservation of the identified miRNAs with those of other plant species.

**Table S2** 86 miRNAs identified here that were conserved with those of N. tabacum without mismatch in sequence.

**Table S3** Primers used for experiments.
