# Supplementary material for: Suppression of nbe‐miR166h‐p5 attenuates leaf yellowing symptoms of potato virus X on Nicotiana benthamiana and reduces virus accumulation
Source: Mol Plant Pathol. 2018 Sep 28;19(11):2384–96. doi: 10.1111/mpp.12717 (PMC6638021; doi:10.1111/mpp.12717)
Supplement: Supplementary file 1 — Fig. S1 Sequence alignment between the precursors of ntamiR166h and nbe‐miR166h. [file MPP-19-2384-s001.docx]

Fig. S1 Sequence alignment between the precursors of nta-miR166h and nbe-miR166h.
