# Supplementary material for: Suppression of nbe‐miR166h‐p5 attenuates leaf yellowing symptoms of potato virus X on Nicotiana benthamiana and reduces virus accumulation
Source: Mol Plant Pathol. 2018 Sep 28;19(11):2384–96. doi: 10.1111/mpp.12717 (PMC6638021; doi:10.1111/mpp.12717)
Supplement: Supplementary file 2 — Fig. S2 The expression of nbe‐miR166h was not affected in VbMS‐treated plants. [file MPP-19-2384-s002.docx]

Fig. S2 The expression of nbe-miR166h was not affected in VbMS-treated plants.
