# Supplementary material for: Suppression of nbe‐miR166h‐p5 attenuates leaf yellowing symptoms of potato virus X on Nicotiana benthamiana and reduces virus accumulation
Source: Mol Plant Pathol. 2018 Sep 28;19(11):2384–96. doi: 10.1111/mpp.12717 (PMC6638021; doi:10.1111/mpp.12717)
Supplement: Supplementary file 3 — Fig. S3 Plants with silenced targets showed no obvious phenotype and had no significant differences in chlorophyll content. [file MPP-19-2384-s003.docx]

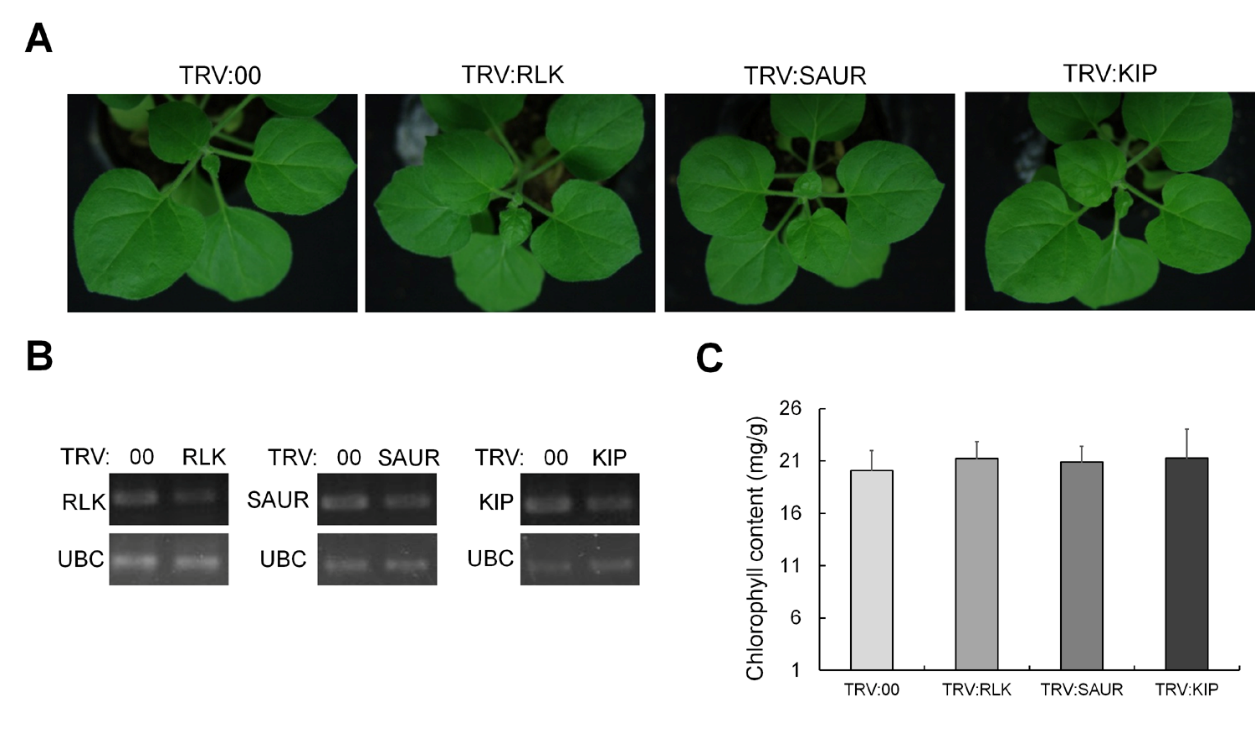


Fig. S3 Plants with silenced targets showed no obvious phenotype and had no significant differences in chlorophyll content.
